# Prognostic value of immune factors in the tumor microenvironment of patients with pancreatic ductal adenocarcinoma

**DOI:** 10.1186/s12885-021-08911-4

**Published:** 2021-11-10

**Authors:** Sachie Kiryu, Zensho Ito, Machi Suka, Tsuuse Bito, Shin Kan, Kan Uchiyama, Masayuki Saruta, Taigo Hata, Yuki Takano, Shuichi Fujioka, Takeyuki Misawa, Takashi Yamauchi, Hiroyuki Yanagisawa, Nobuhiro Sato, Toshifumi Ohkusa, Haruo Sugiyama, Shigeo Koido

**Affiliations:** 1 Division of Gastroenterology and Hepatology, Department of Internal Medicine, The Jikei University School of Medicine, Kashiwa Hospital, 163-1 Kashiwa-shita, Kashiwa, Chiba, 277-8567 Japan; 2 Department of Public Health and Environmental Medicine, The Jikei University School of Medicine, 3-25-8 Nishi-shimbashi, Minato-ku, Tokyo, 105-8461 Japan; 3 Institute of Clinical Medicine and Research, The Jikei University School of Medicine, 163-1 Kashiwa-shita, Kashiwa, Chiba, 277-8567 Japan; 4 Division of Gastroenterology and Hepatology, Department of Internal Medicine, The Jikei University School of Medicine, 3-25-8 Nishi-shimbashi, Minato-ku, Tokyo, 105-8461 Japan; 5 Department of Surgery, The Jikei University School of Medicine, Kashiwa Hospital, 163-1 Kashiwa-shita, Kashiwa, Chiba, 277-8567 Japan; 6 Department of Microbiota Research, Juntendo University Graduate School of Medicine, 3-3-1 Hongo, Bunkyo-ku, Tokyo, 113-0033 Japan; 7 Department of Functional Diagnostic Science, Osaka University Graduate School of Medicine, Suita-city, Osaka, 565-0871 Japan

**Keywords:** Foxp3, Immune-related cells, Pancreatic cancer, Prognosis, PD-1, TIL, Surgical resection

## Abstract

**Background:**

Both activated tumor-infiltrating lymphocytes (TILs) and immune-suppressive cells, such as regulatory T cells (Tregs), in the tumor microenvironment (TME) play an important role in the prognosis of patients with pancreatic ductal adenocarcinoma (PDAC).

**Methods:**

The densities of TILs, programmed death receptor 1 (PD-1) + T cells, and forkhead box P3 (Foxp3) + T cells were analyzed by immunohistochemical staining. The associations of the immunological status of the PDAC microenvironment with overall survival (OS) time and disease-free survival (DFS) time were evaluated.

**Results:**

PDAC patients with a high density of TILs in the TME or PD-1-positive T cells in tertiary lymphoid aggregates (TLAs) demonstrated a significantly better prognosis than those with a low density of TILs or PD-1-negativity, respectively. Moreover, PDAC patients with high levels of Foxp3-expressing T cells showed a worse prognosis than those with low levels of Foxp3-expressing T cells. Importantly, even with a high density of the TILs in TME or PD-1-positive T cells in TLAs, PDAC patients with high levels of Foxp3-expressing T cells showed a worse prognosis than patients with low levels of Foxp3-expressing T cells. A PDAC TME with a high density of TILs/high PD-1 positivity/low Foxp3 expression was an independent predictive marker associated with superior prognosis.

**Conclusion:**

Combined assessment of TILs, PD-1+ cells, and Foxp3+ T cells in the TME may predict the prognosis of PDAC patients following surgical resection.

**Supplementary Information:**

The online version contains supplementary material available at 10.1186/s12885-021-08911-4.

## Background

Pancreatic ductal adenocarcinoma (PDAC) is a solid tumor with a highly immunosuppressive tumor microenvironment (TME) and is considered a highly lethal disease [1]. PDAC is characterized by an extensive dense desmoplastic stroma including numerous types of cells, such as fibroblasts, effector T cells, and immunosuppressive cells (regulatory T cells (Tregs), myeloid-derived suppressor cells (MDSCs), and tumor-associated macrophages (TAMs)), all of which interact with tumor cells [2, 3]. The interaction of tumor cells with immune factors in the TME suppresses antitumor immune reactions, resulting in tumor progression and metastasis [4, 5]. Therefore, PDAC is considered to be an “immunologically cold” tumor filled with many types of immunosuppressive cells and a limited number of effector T cells.

A high density of PD-1+ T cells in the TME is known to be related to a significantly good prognosis in resected PDAC [6]. In PDAC patients with a high density of PD-1+ T cells, anti-programmed death receptor 1 (PD-1)/programmed death ligand 1 (PD-L1) therapy may improve the antitumor immune response [6–9]. Another mechanism underlying the prognosis of PDAC is apparently the dominance of immunosuppressive cells such as Tregs in the TME [10–15]. Forkhead box P3 (Foxp3) is highly expressed in Tregs and has been identified as a key player in the function of Tregs [16]. Infiltrating Foxp3+ Tregs in the TME suppress the function of effector T cells and dendritic cells (DCs) by secreting suppressive cytokines, such as IL-10 and tumor growth factor (TGF)-β, or through the cell-mediated engagement of inhibitory receptors [11].

Poor prognosis in PDAC is partially attributed to the complex interactions in an “immunologically cold” TME [10]. Therefore, both infiltration of immunosuppressive cells and lack of activated tumor-infiltrating lymphocytes (TILs) in the PDAC TME may be closely associated with poor outcomes. Identifying markers associated with the prognosis of postoperative PDAC patients is important for improving their outcomes. Previously, we analyzed TILs in 63 consecutive PDAC patients who underwent R0 resection between January 2005 and December 2017 [3]. Thereafter, 22 consecutive PDAC patients underwent R0 resection between January 2018 and March 2019. Therefore, in this study, the clinical significance of TILs, PD-1+ T cells, and Foxp3+ T cells in the TME of 85 PDAC patients was comprehensively analyzed to determine the prognostic implications of immune-related cells.

## Methods

### PDAC patients

Between January 2005 and March 2019, 85 consecutive PDAC patients (47 males, 38 females; median age, 67 years) who underwent R0 resection at Jikei University Kashiwa Hospital were enrolled in this study. The 85 PDAC patients included 63 patients in whom TILs (CD3, CD4, and CD8 expression) had been analyzed by semiquantitative scores of the 3 different tumor areas in a previous study [3]. In this study, we analyzed the association of TILs and other immune-related factors, including PD-1+ T cells and Foxp3+ T cells by counting cell numbers from 10 random tumor areas, using increased PDAC patient numbers. Overall survival (OS) was defined as the time from diagnosis to the time of cancer death. Disease-free survival (DFS) was defined as the time from surgery to the first evidence of objective cancer progression.

### Immunohistochemical staining [1, 3, 8, 14]

Samples were fixed in 10% buffered formalin for 1 day at room temperature, and paraffin-embedded sections from surgically resected PDAC samples were sliced into 4 μm thick sections and placed on glass slides. Antigen retrieval for PD-1 or Foxp3 staining was performed by heating the sections for 12 min at 110 °C with Target Retrieval Solution (Citrate buffer, pH 6.0; Dako: Agilent Technologies Inc., Santa Clara, CA, USA) or Target Retrieval Solution (Tris/EDTA buffer, pH 9.0; Dako: Agilent Technologies Inc.), respectively. Antigen retrieval for CD3 and CD4 staining was performed in Histofine Antigen Retrieval Solution (Tris/EDTA buffer, pH 9.0) (Nichirei Bioscience Corporation, Tokyo, Japan) at 110 °C for 12 min. For CD8 staining, samples were incubated in Target Retrieval Solution (Citrate buffer, pH 6.0; Dako: Agilent Technologies Inc.) at 110 °C for 12 min. Endogenous peroxidase activity was blocked with a 3% H_2_O_2_ solution (Wako Pure Chemical Industries, Ltd., Osaka, Japan) at room temperature for 10 min. Then, the slides were incubated with a rabbit anti-human PD-1 monoclonal antibody (clone D4W2J; dilution 1:100; Cell Signaling Technology Inc., Danvers, MA, USA) or a mouse anti-human Foxp3 antibody (clone 236A/E7; dilution 1:200; Thermo Fisher Scientific Inc., Waltham, MA, USA) overnight at 4 °C or a mouse anti-human CD3, CD4, or CD8 monoclonal antibody (clone PS1, 4B12, and C8/144B, respectively; Nichirei Bioscience Corporation, Tokyo, Japan) for 30 min at room temperature. To detect these antigens by light microscopy, the EnVision detection system (Dako: Agilent Technologies Inc.) containing horseradish peroxidase-conjugated mouse/rabbit IgG and a 3,30-diaminobenzidine-peroxidase solution was used. In addition, nuclei were counterstained with hematoxylin. Negative control staining was applied as follows: a mouse IgG monoclonal antibody (Santa Cruz Biotechnology Inc. Santa Cruz, CA, USA) for TILs (CD3, CD4, or CD8), a mouse IgG1 monoclonal antibody (G3A1: CST Inc.) for Foxp3, and a rabbit IgG monoclonal antibody (DA1E; CST Inc., Danvers, MA, USA) for PD-1.

### Immunohistochemical assessment of immune-related cells

Cells immunolabeled with antibodies specific for CD3, CD4, CD8, or Foxp3 were evaluated by three observers who were unaware of the clinical information of the PDAC patients. The densities of TILs in the PDAC TME regions were evaluated by counting the number of stained cells from 10 random tumor areas under × 400 magnification. Then, immune-related cell densities were graded as low (≤ median cell counts/mm^2^ tumor area) or high (> median cell counts/mm^2^ tumor area). Unlike TILs, PD-1+ cells were not detected in many PDAC patients and were mostly detected in tertiary lymphoid aggregates (TLAs). Therefore, PD-1 expression was assessed as positive or negative in TLAs.

### Clinicopathological data

The clinicopathological characteristics of the PDAC patients were retrospectively analyzed from the patients’ medical records and included age, sex, and histological features (location, differentiation, and size) and stage by the American Joint Committee on Cancer (AJCC) [17].

### Laboratory data

Laboratory data, including peripheral blood lymphocyte, monocyte, neutrophil, and platelet counts, hemoglobin, C-reactive protein (CRP), albumin (Alb), lactate dehydrogenase (LDH), amylase (AMY), and tumor marker (carcinoembryonic antigen [CEA] and carbohydrate antigen 19–9 [CA19–9]) levels within 2 weeks before surgery, were collected to assess their prognostic impact on patients who underwent PDAC resection. Moreover, systemic inflammatory nutritional factors, the neutrophil/lymphocyte ratio (NLR), platelet/lymphocyte ratio (PLR), lymphocyte/monocyte ratio (LMR), CRP/Alb ratio (CAR), and Glasgow prognostic score (GPS) were also evaluated before surgery. In PDAC patients, the GPS was evaluated as follows: score 2, elevated CRP level (> 1.0 mg/dL) and hypoalbuminemia (Alb < 3.5 g/dL); score 1, either elevated CRP level (> 1.0 mg/dL) or hypoalbuminemia (Alb < 3.5 g/dL); and score 0, neither elevated CRP level (> 1.0 mg/dL) or hypoalbuminemia (Alb < 3.5 g/dL).

### Statistical analysis [3]

Immune-related cells (cells with CD3, CD4, CD8, PD-1, or Foxp3 positivity) and laboratory data were assessed for significant prognostic value. The Kaplan-Meier method was used to estimate OS and DFS times, and the differences between the groups were compared with the log-rank test. Univariate and multivariate analyses were performed using Cox proportional hazard models to evaluate the influence of multiple parameters, including lymphocyte numbers and NLR and GPS as continuous variables, as independent predictors of OS and DFS times. Hazard ratios (HRs) with 95% confidence intervals (CIs) were calculated using Cox proportional hazard models. Differences in clinicopathological characteristics or laboratory parameters between groups of patients with different immune-related cell densities were evaluated using the Mann-Whitney U test. The χ^2^ test was used to determine the association between PDAC patient characteristics and immune-related cells. *P* < 0.05 was considered to indicate a statistically significant difference. The statistical analyses were conducted using SAS version 9.4 software (SAS Institute, Cary, NC, USA).

## Results

### PDAC patient characteristics

A total of 85 consecutive patients who underwent PDAC resection (R0) between January 2005 and March 2019 were analyzed. The clinical characteristics of the PDAC patients are summarized in Table 1. There were 47 (55.29%) male and 38 (44.71%) female patients. The median patient age at diagnosis was 67 years (range, 33–85 years). PDAC lesions were located in the head of the pancreas (75.29%). The numbers of PDAC patients with AJCC stage I/II and III disease were 59 (69.41%) and 26 (30.59%), respectively. Sixty-eight out of 85 (80.00%) PDAC patients received chemotherapy once recurrence was identified. The median OS and DFS times of the PDAC patients following surgical resection were 528 and 284 days, respectively, among the 85 enrolled PDAC patients. As the follow-up time for all PDAC patients was more than two years, no patients were alive at the time of analysis in this study.

**Table 1:** Clinicopathological characteristic of patients with pancreatic ductal adenocarcinoma

All cases	n	%
	85	100%
Age at surgery		
< median (67-years old)	37	43.53
≥ median	48	56.47
Sex		
Male	47	55.29
Female	38	44.71
Tumor location		
Head	64	75.29
Body-to-Tail	21	24.71
Tumor differentiation		
Well-to-Moderate	71	83.53
Poor	14	16.47
Tumor stage		
I and II	59	69.41
III	26	30.59
Tumor size		
< median (35 mm)	40	47.09
≥ median	45	52.94

### Laboratory data

The baseline laboratory parameters obtained within 2 weeks before surgery, such as leukocyte, lymphocyte, monocyte, neutrophil, and platelet counts and hemoglobin, CRP, Alb, LDH, AMY, CEA, and CA19–9 levels, are shown in supplemental Table 1. Moreover, systemic inflammatory nutritional factor-based parameters such as the NLR, PLR, LMR, CAR, and GPS were also evaluated (Table S1).

### Associations of TILs with OS and DFS times

**Fig. 1:**
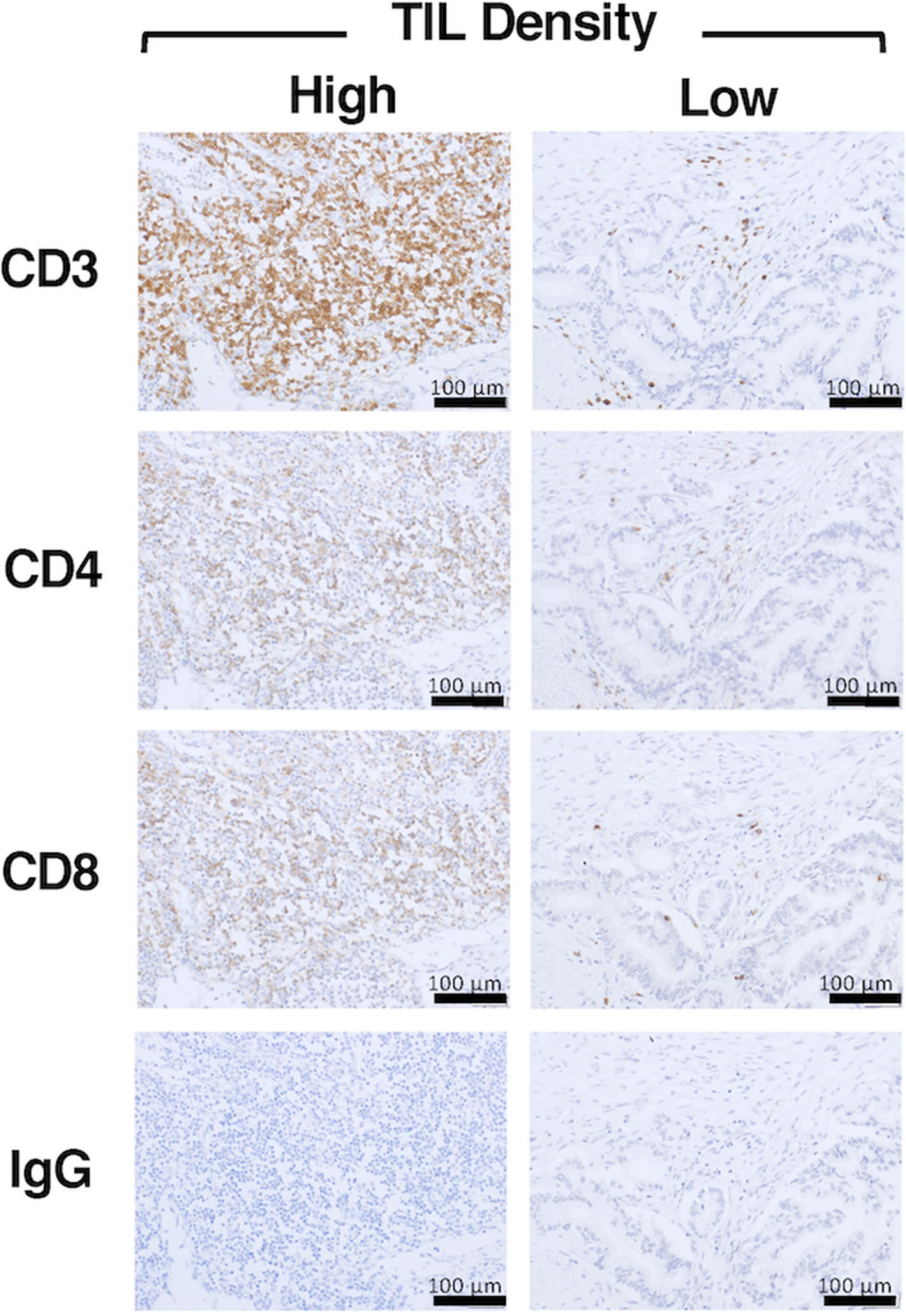
Immunohistochemical staining of TILs in samples from PDAC patients following surgical resection. Quantitative scoring of the TILs surrounding ten random tumor areas in PDAC samples was performed. The average of cell counts/mm^2^ tumor area was graded as low density (≤ median cell counts/mm^2^ tumor area) or high density (> median cell count/mm^2^ tumor area). Negative control samples were stained with a nonspecific IgG antibody. PDAC, pancreatic ductal adenocarcinoma; TIL, tumor-infiltrating lymphocyte; IgG: immunoglobulin G

**Fig. 2:**
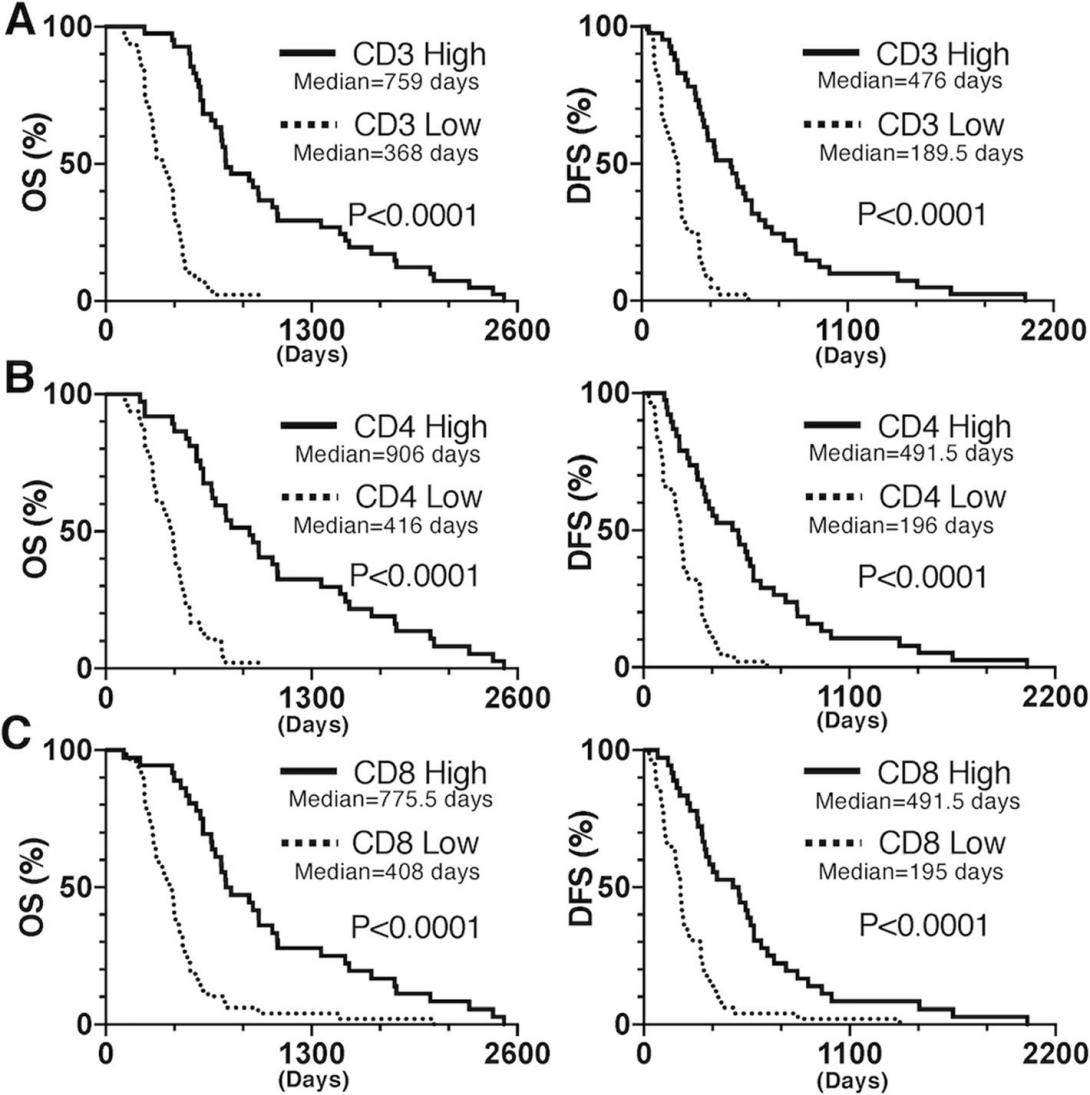
Associations of TIL densities with OS and DFS times in PDAC patients following surgical resection. Kaplan-Meier survival curves for TILs in PDAC patients following surgical resection. PDAC patients were classified into 2 subgroups: a group with a high density and a group with a low density of TILs. The Kaplan-Meier estimates of OS (left panel) and DFS (right panel) time in the 2 subgroups according to the densities of CD3+ (A), CD4+ (B), or CD8+ (C) T cells are shown. PDAC: pancreatic ductal adenocarcinoma; OS: overall survival; DFS: disease-free survival

TILs in a wide range of human cancers play an essential role in eradicating tumor cells [18–20]. Therefore, to analyze the densities of different T cell subsets in the TME and their prognostic implications, we performed immunohistochemical staining to determine the levels of CD3, CD4, and CD8 in the TME. The density of TILs has been proposed as an independent predictor of prognosis in several types of solid tumors [3, 10, 19, 20]. In line with these observations, TIL (CD3+, CD4+, and CD8+ T cell) density in 85 PDAC patients who underwent surgical resection was analyzed first. We previously assessed a TIL density in 65 PDAC patients by a semiquantitative scoring system from 3 tumor areas [3]. Therefore, to avoid some bias of the results in this study, 85 PDAC patients including these 65 patients were analyzed by counting TIL numbers from 10 random tumor areas. The median of average cell numbers in CD3+, CD4+ and CD8+ T cells was 942.5, 153.1, and 111.2 /mm^2^ tumor area, respectively. PDAC patients were divided into two 2 groups: a group with high TIL density (> median cell counts/mm^2^ tumor area) and a group with low TIL density (≤ median cell counts/mm^2^ tumor area) (Fig. 1). Then, we performed Kaplan-Meier survival analyses based on CD3, CD4, and CD8 expression between the groups with different TIL densities and observed a significant association of high CD3+, CD4+, and CD8+ T cell density with increased OS (median OS of 759, 906, and 775.5 days vs. median OS of 368, 416, and 408 days for low CD3+, CD4+, and CD8+ T cell expression, respectively; *p* < 0.0001) (Fig. 2A, B, and C). Similar results were observed for DFS: the median DFS values of PDAC patients with high CD3+, CD4+, and CD8+ TIL densities (476, 491.5, and 491.5 days, respectively) were significantly higher than those of patients with low densities (189.5, 196, and 195 days, respectively; p < 0.0001) (Fig. 2A, B, and C). In short, high TIL density in the TME had significant associations with increased OS and DFS in PDAC patients following surgical resection.

### Associations of PD-1 in the PDAC TME with OS and DFS times

**Fig. 3:**
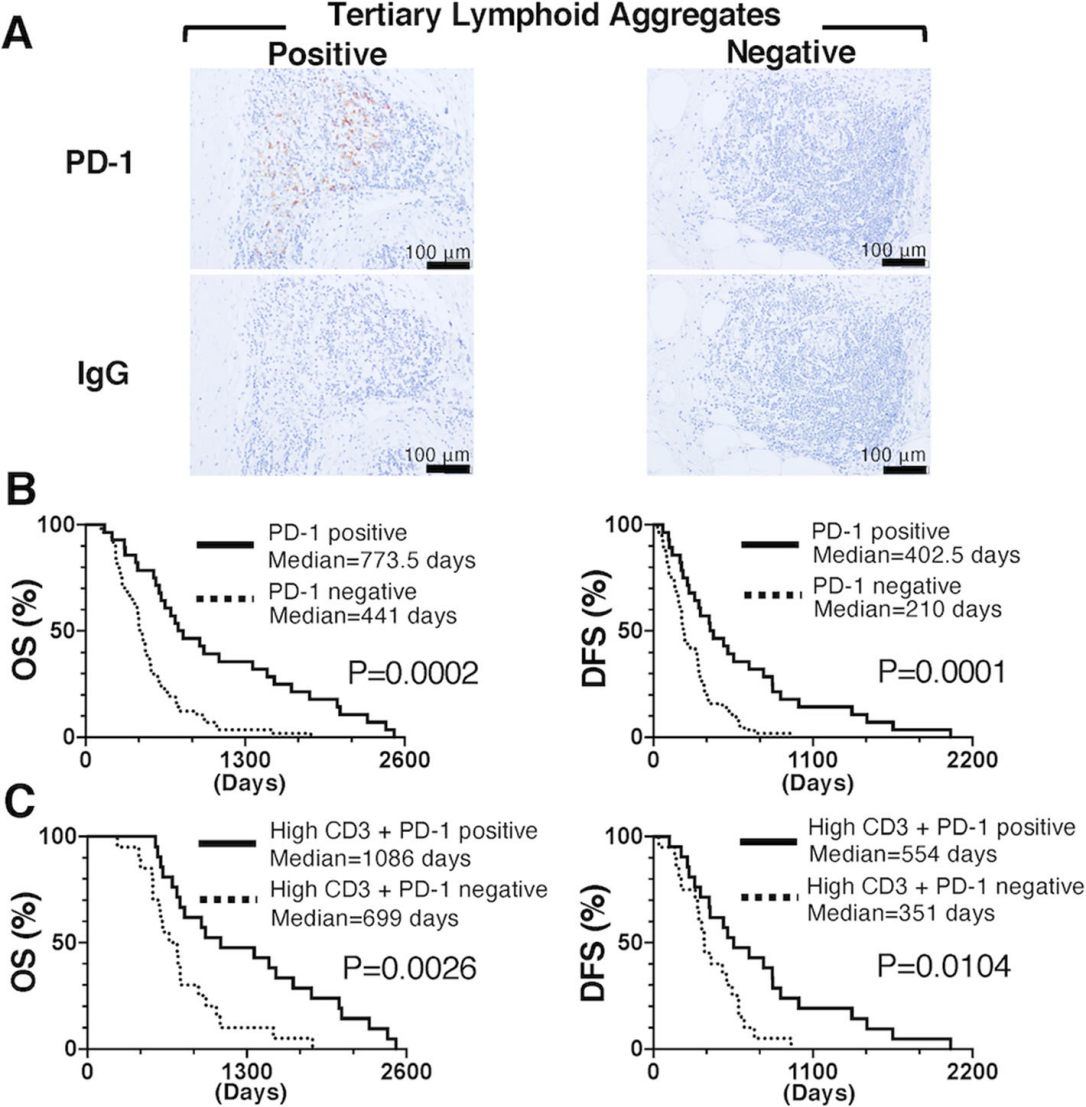
Association of PD-1+ cells in tertiary lymphoid aggregates with clinical prognosis. PD-1 expression was mainly observed in tertiary lymphoid aggregates, and samples were classified into 2 subgroups: PD-1 positive and negative (**A**). The Kaplan-Meier estimates of the OS (left panel) and DFS (right panel) times of the 2 subgroups are shown (**B**). PDAC patients with a high CD3+ T cell density were classified into 2 subgroups: high CD3+/PD-1 positive and high CD3+/PD-1 negative. The Kaplan-Meier estimates of the OS (left panel) and DFS (right panel) times of the 2 subgroups are shown (**C**). PD-1: programmed death 1; PDAC: pancreatic ductal adenocarcinoma; OS: overall survival; DFS: disease-free survival

PD-1 is upregulated at the activated TIL surface through activation of the TCR in naive T cells [21–23]. Therefore, we next assessed the prognostic value in terms of associations with OS and DFS of PD-1 expression in the TME by immunohistochemical staining. PD-1 was only expressed in cell membranes of TILs. Moreover, PD-1+ cells were mainly detected in TLAs in the PDAC TME (Fig. 3A). Therefore, PDAC patients were divided into two groups: PD-1-positive (*n* = 28) and PD-1 negative (*n* = 57) TLAs. Kaplan-Meier survival analysis revealed that PDAC patients with PD-1-positive TLAs had significantly superior OS (positive vs. negative: mean 773.5 vs. 441 days; *p* = 0.0002) (Fig. 3B) and DFS (positive vs. negative: mean 402.5 vs. 210 days; *p* = 0.0001) (Fig. 3B).

### Association between TIL density combined with PD-1 and clinical prognosis

Our data demonstrated the clinical impact of TILs and PD-1 expression as independent factors on the OS and DFS of PDAC patients following surgical resection. Next, we also assessed the prognostic value of a combined immunological status of high TILs and PD-1 expression in TLAs in the PDAC TME. Generally, TILs can be detected as CD3+ T cells in the TME. Therefore, PDAC patients with high CD3+ T cells were divided into four groups based on CD3+ T cell density and PD-1 expression in TLAs: high CD3+ T cells/PD-1-positive TLAs, high CD3+/PD-1-negative TLAs, low CD3+ T cells/PD-1-positive TLAs, and low CD3+ T cells/PD-1-negative TLAs. The subgroup analysis demonstrated that the group with high CD3+ T cells/PD-1-positive TLAs (*n* = 21) had a significantly better prognosis (median OS: 1086 days; median DFS: 554 days) than the group with high CD3+ T cells/PD-1-negative TLAs (*n* = 20) (median OS: 699 days and median DFS: 351 days) (*p* = 0.0026 and 0.0104, respectively) (Fig. 3C). Significant differences in median OS and DFS values in the group with high CD4+ T cells/PD-1-positive TLAs (*n* = 19) (median OS: 1360 days and median DFS: 554 days) (Fig. S1A) and the group with high CD8+ T cells/PD-1-positive TLAs (*n* = 18) (median OS: 1223 days and median DFS: 607 days) compared with the respective groups with PD-1-negative TLAs were also observed (Fig. S1B). In addition, there was no difference between PDAC patients with low CD3+ T cells/PD-1-positive TLAs (*n* = 7) (median OS: 320 days and median DFS: 184 days) and those with low CD3+ T cells/PD-1-negative TLAs (*n* = 37) (median OS: 368 days and median DFS: 195 days) (data not shown). These results indicate that PDAC patients with high CD3+, CD4+, or CD8+ cell density and PD-1-expressing TLAs have a better prognosis than those with low CD3+, CD4+, or CD8+ cell density and PD-1-expressing TLAs.

### Associations of Foxp3 density in the PDAC TME with OS and DFS times

**Fig. 4:**
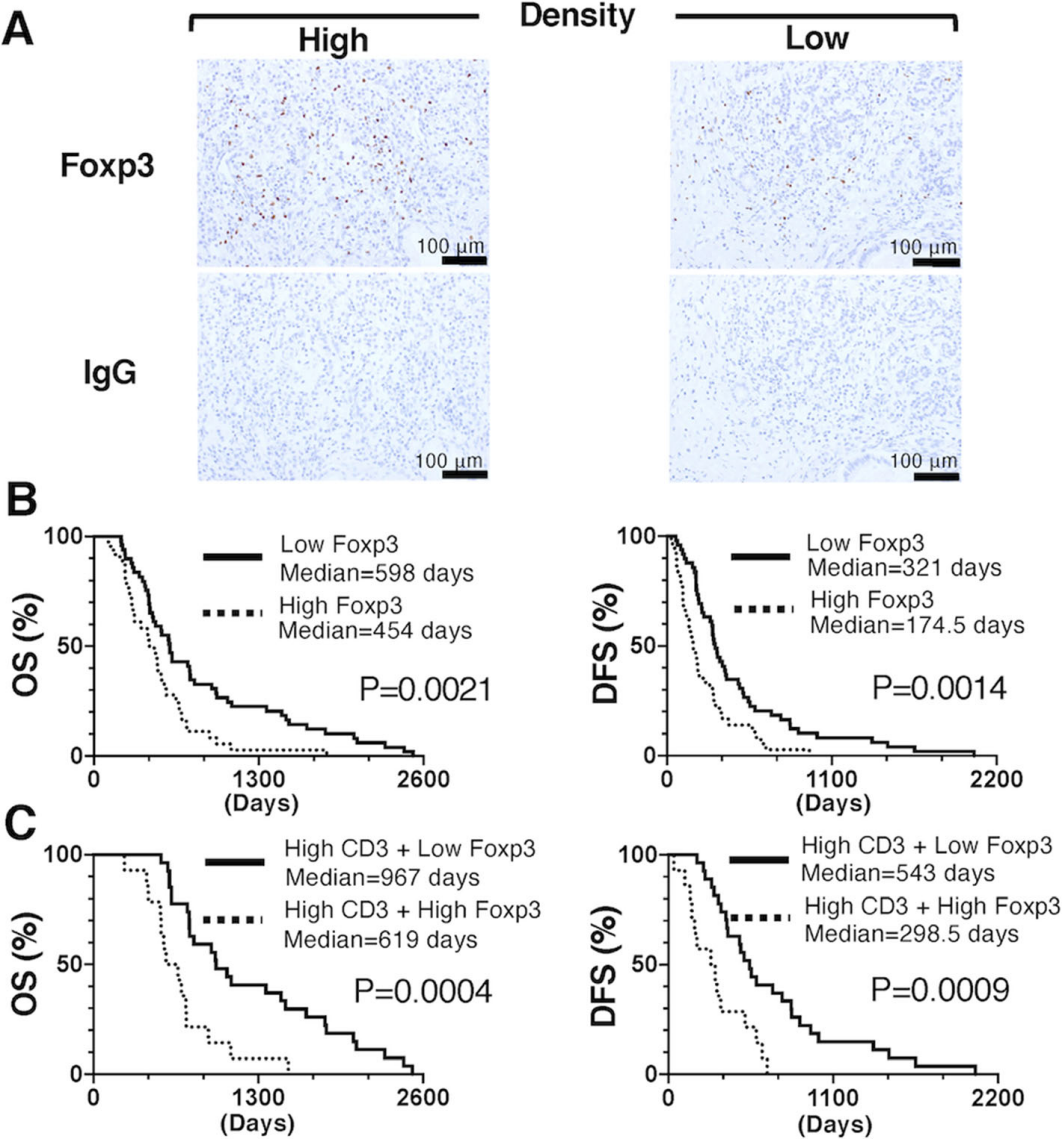
Association of Foxp3+ T cells in the PDAC TME with clinical prognosis. Quantitative scoring of the Foxp3+ T cells surrounding 10 random tumor areas in PDAC samples was performed. The average counts of cells/mm^2^ tumor area were graded as low density (≤ median cell counts/mm^2^ tumor area) or high density (> median cell counts/mm^2^ tumor area) (**A**). Samples were classified into 2 subgroups based on Foxp3+ T cell density: high and low Foxp3+ cell density. The Kaplan-Meier estimates of the OS (left panel) and DFS (right panel) times of the 2 subgroups are shown (**B**). PDAC patients with high CD3+ T cell density were classified into 2 subgroups: high CD3+/high Foxp3+ T cell density and high CD3+/low Foxp3+ T cell density. The Kaplan-Meier estimates of the OS (left panel) and DFS (right panel) times of the 2 subgroups are shown (**C**). Foxp3: forkhead box P3; PDAC: pancreatic ductal adenocarcinoma; OS: overall survival; DFS: disease-free survival

There is increasing evidence suggesting that Foxp3+ Tregs inhibit antitumor immune responses and thus result in poor clinical outcomes in several types of tumors [24]. Therefore, to understand how immunosuppressive cells such as Tregs in the TME of PDAC are associated with clinical benefits, Foxp3+ T cell density was assessed by immunohistochemical staining. The average of Foxp3+ T cell numbers from 10 random tumor areas was 48.5 /mm^2^. Then, 85 PDAC patients were divided into two groups: a group with high (> median cell counts/mm^2^ tumor area) and a group with low (≤ median cell counts/mm^2^ tumor area) (Fig. 4A). Kaplan-Meier survival analysis revealed that PDAC patients with a high density of Foxp3+ T cells (*n* = 36) had a significantly poorer prognosis (median OS (454 days) and DFS (174.5 days)) than those with a low density of Foxp3+ T cells (*n* = 49; median OS (598 days) and DFS (321 days) (OS: *p* = 0.0021 and DFS: *p* = 0.0014) (Fig. 4B). These results indicate that high Foxp3+ T cell density is a factor associated with poor OS and DFS in PDAC patients following surgical resection.

### Association between TILs combined with Foxp3 density and clinical prognosis

We next assessed the prognostic value of TILs combined with Foxp3+ T cell density. CD3+ T cells were again considered to represent TILs in the TME in this analysis. Therefore, PDAC patients were divided into the following four subgroups based on CD3+ and Foxp3+ T cell densities: high CD3+/high Foxp3+ T cells, high CD3+/low Foxp3+ T cells, low CD3+/high Foxp3+ T cells, and low CD3+/low Foxp3+ T cells. The median OS and DFS of the group with high CD3+/low Foxp3+ T cells (*n* = 27) (967 and 543 days, respectively) were significantly superior to those of the group with high CD3+/high Foxp3+ T cells (*n* = 14) (619 and 298.5 days, respectively) (*p* = 0.0004 and 0.0009, respectively) (Fig. 4C). Moreover, significant differences were also observed in the median OS and DFS between groups with different densities of CD4+ (Fig. S2A) and CD8+ (Fig. S2B) T cells. In addition, there was no difference in prognosis between PDAC patients with low CD3+/high Foxp3+ T cells (*n* = 22) (median OS: 308.5 days and median DFS: 135 days) and those with low CD3+/low Foxp3+ T cells (n = 22) (median OS: 404 days and median DFS: 198 days) (data not shown). These results indicate that the clinical prognosis of PDAC patients with high CD3+, CD4+, or CD8+ T cell densities with low Foxp3+ T cell density was better than that of patients with high CD3+, CD4+, or CD8+ T cell densities with high Foxp3+ T cell density.

### Association of CD3+ T cell density combined with PD-1+ and Foxp3+ T cell density with clinical prognosis

**Fig. 5:**
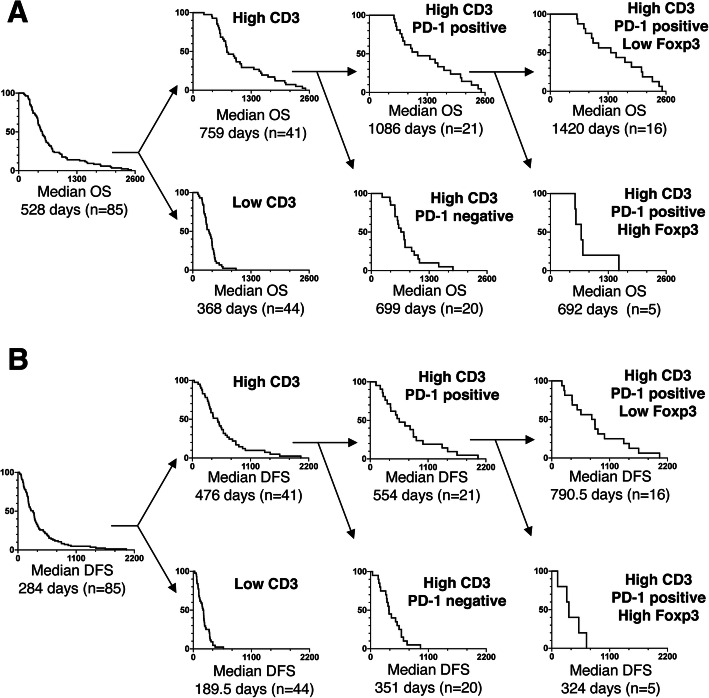
Associations of immune-related cell (CD3+, PD-1+, and Foxp3+ cell) density with clinical prognosis. PDAC samples were classified into 4 subgroups: low CD3+ T cell density, high CD3+ T cell density/PD-1-negative T cells, high CD3+ T cell density/PD-1-positive T cells/low Foxp3+ T cell density, and high CD3+ T cell density/PD-1-positive T cells/high Foxp3+ T cell density. The Kaplan-Meier estimates of OS (A) or DFS (B) time are shown. PD-1: programmed death 1; Foxp3: forkhead box P3; PDAC: pancreatic ductal adenocarcinoma; OS: overall survival; DFS: disease-free survival

PDAC patients with a high CD3+ T cell density, high PD-1+ T cell density, or low Foxp3+ T cell density have a better prognosis (in terms of OS and DFS) than other patients. Therefore, we next assessed the association between clinical prognosis and immune-related cells (CD3+ T cell density combined with PD-1+ and Foxp3+ T cell density). First, PDAC patients with high CD3+ T cell density were divided into two subgroups: high CD3+ T cell density/PD-1-positive TLAs and high CD3+ T cell density/PD-1-negative TLAs. Then, the group with high CD3+ T cell density/PD-1-positive TLAs was divided into the following two subgroups: high CD3+/PD-1 positive/low Foxp3+ T cells and high CD3+/PD-1 positive/high Foxp3+ T cells. As shown in Fig. 5, the Kaplan-Meier survival analysis revealed that the OS and DFS in the group with high CD3+/PD-1-positive/low Foxp3+ T cells (*n* = 16) (1420 days and 790.5 days, respectively) were significantly better than those in the group with high CD3+/PD-1-positive/high Foxp3+ T cells (*n* = 5) (692 days and 324 days, respectively) (*p* = 0.0093 and 0.0249, respectively). These results indicate that the group with high CD3+/PD-1-positive/low Foxp3+ T cells had the best prognosis.

### Univariate and multivariate analyses of clinical prognosis

Some laboratory parameters (lymphocyte counts and CA19–9 levels) and inflammatory nutritional factors, such as the NLR and GPS, have been shown to be prognostic factors for several types of cancer patients [25–27]. As CA19–9 levels within 2 weeks before surgery were not available in some cases, we evaluated age, histological features (location, differentiation, and size), lymphocyte counts, NLR, and GPS as clinical prognostic factors. Moreover, local tumor immune-related cells (CD3+, CD4+, CD8+, PD-1+, and Foxp3+ T cells) were also evaluated. As shown in Tables S2 and S3, age, histological features (location and size), lymphocyte counts, NLR, and GPS were not identified as significant prognostic factors by univariate and multivariate analysis. However, tumor differentiation was an independent predictor of survival (OS and DFS) after surgical resection by univariate analysis (*p* = 0.047 and 0.023, respectively). Interestingly, high TIL (CD3+, CD4+, or CD8+ T cell) density, PD-1-positive TLAs, and low Foxp3+ T cell density were significant independent prognostic factors identified by the multivariate analysis (Tables S2 and S3). Therefore, multivariate Cox proportional hazard analysis was further performed to compare the association with clinical prognosis (in terms of OS and DFS) in four groups: low CD3+ T cell density, high CD3+ T cell density/PD-1-negative T cells, high CD3+ T cell density/PD-1-positive T cells/high Foxp3+ T cell density, and high CD3+ T cell density/PD-1-positive T cells/low Foxp3+ T cell density. The HRs (95% CIs) across categories (high CD3+ T cell density/PD-1-negative T cells, high CD3+ T cell density/PD-1-positive T cells/high Foxp3+ T cell density, and high CD3+ T cell density/PD-1-positive T cells/low Foxp3+ T cell density) for OS were 0.136, 0.139, and 0.035, respectively (reference 1.00), with a *p* value trend of < 0.001 (Table S2), and for DFS, the HRs were 0.245, 0.326, and 0.065 (reference 1.00), with a p value trend of < 0.001, 0.028, and < 0.001, respectively) (Table S3). These results suggested that the high CD3+ T cell density/PD-1-positive T cells/low Foxp3+ T cell density combination was the best independent prognostic factor identified by multivariate Cox proportional hazards regression analysis.

### Associations of patient characteristics with immune-related cells

The associations of clinicopathological parameters (age, sex, and tumor characteristics (location, differentiation, stage, and size)) of PDAC patients with immune-related cell (CD3+, CD4+, CD8+, PD-1+, and Foxp3+ cell) densities are illustrated in Table S4. There were no statistically significant associations between clinicopathological parameters and immune-related cell (CD4+, CD8+, PD-1+, and Foxp3+ cell) densities in the TME in PDAC.

### Associations of laboratory parameters with immune-related cell (TIL, PD-1+, and Foxp3+ cell) infiltration

We also assessed the associations of laboratory parameters (leukocyte, lymphocyte, monocyte, neutrophil, and platelet counts, hemoglobin, CRP, Alb, LDH, AMY, CEA, and CA19–9 levels) as well as systemic inflammatory nutritional markers (NLR, PLR, LMR, CAR, and GPS) with immune-related cell (CD3+, CD4+, CD8+, PD-1+, and Foxp3+ cell) densities in the TME in PDAC. There were no statistically significant associations between laboratory parameters and immune-related cell densities in the TME in PDAC (Table S5). However, high GPS was significantly observed in PDAC patients with low CD4+ T cell density, compared to high CD4+ T cell density (*p* = 0.026).

### Association of immune-related cells with clinical prognosis in PDAC patients with stage I/II or III disease

Since R0 patients included those with stage I, II, and III disease, all data were adjusted to AJCC stages. In this study, the number of PDAC patients with stage I, II, or III disease was 5, 54, or 26, respectively. The frequency of PDAC patients with stage I disease was extremely low (5.88%). Therefore, we divided the patients into 2 groups: stage I/II (*n* = 59) and III (*n* = 26). There was no difference in OS (*p* = 0.7940) or DFS (*p* = 0.4865) between the patients with stage I/II disease and those with stage III disease by log-rank test. In PDAC patients with either stage I/II disease (Table S6) or stage III disease (Table S7), there was a significant difference in OS between high and low TIL (CD3+, CD4+, CD8+ cell) densities. In PD-1 expression in TLAs or Foxp3+ T cell density analysis, there was also a significant difference in OS in PDAC with stage I/II disease but not stage III disease. Moreover, stage I/II PDAC patients with a high CD3+ T cell density, PD-1-positive TLAs or a low density of Foxp3+ T cells showed significantly prolonged OS or DFS (Table S6). In patients with either stage I/II or III disease, PD-1-positive/CD3 high-density PDAC patients who had a low density of Foxp3+ T cells showed a better clinical prognosis (OS or DFS), but the difference was not significant. In addition, there was also no significant difference in OS associated with PD-1 expression in TLAs or Foxp3+ T cell density in the small population of stage III PDAC patients analyzed.

## Discussion

PDAC is one of the most aggressive and lethal cancers with a dismal prognosis and is typically refractory to conventional chemotherapy, radiation therapy, and immune checkpoint inhibitors [28]. The mechanism underlying the poor responsiveness of PDAC to these therapies is apparently related to the effects of an immunosuppressive TME, which result in the progression of PDAC [5]. Although previous studies have examined the prognostic impact of TILs or Tregs on the clinical outcome of PDAC [10, 29], the prognostic value of TILs and their association with TILs, PD-1+ T cells, and Foxp3+ T cells remain largely unexplored. Previously, we al so demonstrated the importance of TIL density as a prognostic and predictive marker using 65 PDAC patients [3]. In the present study, 85 PDAC patients were included in 65 patients previously analyzed for TIL by a semiquantitative scoring system from three tumor areas [3]. Therefore, to avoid some bias of the results and more clearly assess them, 85 PDAC patients including these 65 patients were reanalyzed by coun ting TIL numbers from 10 random tumor areas. Moreover, to assess the immunological mechanism underlying the effects on prognosis in detail, the prognostic impact of immune-related cells (TILs, PD-1-positive T cells, and Tregs) was comprehensively assessed in 85 PDAC patients following surgical resection in this study.

As expected, the results demonstrated that high TIL density in the TME was significantly associated with better OS and DFS than low TIL density. High infiltration of CD8+ T cells in the TME might be essential for inducing antitumor immunity in many types of tumors, including PDAC [30]. Moreover, increasing evidence indicates that the activation and infiltration of CD4+ T cells in the TME play a pivotal role in the induction and maintenance of CD8+ cytotoxic T lymphocytes (CTLs), which lead to effective antitumor immunity [30, 31]. Thus, the result that PDAC patients with strong infiltration of both CD4+ and CD8+ T cells had superior clinical outcomes supports the previous studies [31]. Simultaneous infiltration of not only CD8+ T cells but also activated CD4+ cells in the TME might be essential for efficient antitumor immunity.

As PD-1 is mostly expressed on activated effector CD4+ or CD8+ T cells as opposed to other T cells in normal tissues and peripheral blood T lymphocytes (PBLs) [21, 22, 23], we analyzed the infiltration density of PD-1+ T cells by immunohistochemical analysis. PD-1+ T cell numbers were much lower than CD3+ T cell numbers, suggesting that infiltration of activated T cells in the TME may be rare in PDAC patients. These observations are in line with previous studies showing that PDAC has an “immunologically cold” TME [10]. Moreover, PD-1+ T cells were mostly observed in TLAs. Therefore, PDAC patients were divided into two subtypes: patients with PD-1-positive TLAs and patients with PD-1-negative TLAs. The results that PDAC patients with PD-1-positive TLAs had a significantly better prognosis than those with PD-1-negative TLAs support previous studies showing a positive prognostic impact of PD-1+ T cells in patients with head and neck cancer, ovarian cancer, colorectal cancer, melanoma, or lymphoma [6]. As PDAC is mostly an “immunologically cold” tumor, potential strategies that work by converting its “immunologically cold” TME into an “immunologically hot” TME are urgently needed. Increasing evidence indicates that cancer vaccines targeting tumor-associated antigens (TAAs) are effective in activating antigen-specific CTLs, which then infiltrate the TME [30]. Therefore, cancer vaccines may lead to the upregulation of PD-1 on tumor-reactive T cells and contribute to the infiltration of PD-1+ T cells. Indeed, a previous report demonstrated that vaccination of PDAC patients induced the formation of TLAs and activated T cell infiltration [32]. We also reported that a Wilms’ tumor 1 (WT1) peptide vaccine induced WT1-specific TILs in the PDAC TME, resulting in long-term survival [33]. However, continuous activation of T cells by the same antigens may lead to very high levels of PD-1 expression on antigen-specific CD8+ T cells, which can lead to induction of an exhausted state with loss of cytotoxicity [34, 35]. Our previous study also demonstrated that vaccination of PDAC patients with WT1 peptide-pulsed DCs resulted in slight upregulation of PD-1 on WT1-specific CD8+ T cells in the peripheral blood of super-responders [36]. Therefore, a moderate number of infiltrating PD-1+ T cells in TLAs may be associated with good prognosis in PDAC patients.

Tregs might also modulate the composition of the PDAC TME to affect the prognosis of PDAC patients. Tregs are characterized by the expression of the transcription factor Foxp3, which is a suitable marker for their identification [16]. Foxp3+ T cells impair the induction and activation of T cells, thereby compromising tumor killing and promoting tumor progression [12, 15]. Our results showing that the high Foxp3+ T cell subgroup showed a significantly poorer prognosis than the low Foxp3+ T cell subgroup support the findings of previous reports on various cancers, including PDAC [13–15]. Importantly, even PDAC patients with high TIL infiltration showed a poor prognosis when there was strong infiltration of Foxp3+ T cells in the TME. Understanding the prognostic significance of Foxp3+ T cells in the TME may help in the development of effective therapeutic strategies targeting Tregs to enhance the clinical outcomes of patients with PDAC. Both sufficient CTL infiltration and a reduction of Treg infiltration are necessary for improving the prognosis of PDAC patients [37, 38].

Although PDAC patients with high TIL density have significantly prolonged survival, most PDAC patients develop an immunosuppressive TME that restricts the infiltration and/or function of CTLs [1, 3, 39]. In this regard, the multiple regulatory factors associated with immune-related cells may play an essential role in the survival of PDAC cells. As such, we comprehensively assessed 3 kinds of immune-related cell markers, CD3, PD-1, and Foxp3. Of the 85 PDAC patients, the subgroup with both high CD3+ T cell infiltration and PD-1-positive TLAs (21 cases) had a significantly better prognosis than that with high CD3+ T cell infiltration and PD-1-negative TLAs (20 cases). It has been reported that increased levels of both T cells and B cells in the TME can induce the formation of TLAs in PDAC and are associated with favorable prognosis [40, 41]. PDAC patients with increased levels of both T and B cells may have an “immune-rich” TME. In this study, 21 patients with PDAC with an “immune-rich” TME (high CD3+ T cell infiltration with PD-1 positive TLAs) were divided into two subgroups according to the infiltration levels of Foxp3+ T cells. Interestingly, 16 of the 21 PDAC patients with an “immune-rich” TME and a low Foxp3+ T cell density had the best prognosis (OS: 1420 days and DFS: 790.5 days). An “immune-rich” TME may represent an active immune status. However, 5 of the 21 PDAC patients with an “immune-rich” TME exhibited high Foxp3+ T cell density and had a significantly decreased prognosis (OS: 692 days and DFS: 324 days). That is, there was a subgroup of PDAC patients with an “immune-rich” TME and this subgroup had worse outcomes. The results suggest that even though PDAC patients show an “immune-rich” TME, the antitumor effect may be cancelled by Foxp3+ T cells (which are normally present in an “immune-tolerant” TME), resulting in a worse prognosis [42]. Therefore, strategies to increase CTL infiltration and deplete Tregs to increase antitumor immune responses are urgently required.

These observations may suggest that since R0 PDAC patients were from stages I, II, and III, it is necessary that all data be adjusted to AJCC stages [17]. However, the number of PDAC patients with stage I (*n* = 5) or III (*n* = 26) disease was very low. Therefore, it was difficult to compare between two subgroups among PDAC with stage I or III. In PDAC patients with stage I/II disease (*n* = 59), subgroups with high TIL density, PD-1 expression in TLAs, or low Foxp3+ T cell density exhibited significantly prolonged OS. However, it was also difficult to compare between small groups with stage I/II: high Foxp3 density in PD-1 positive/CD3 high density (*n* = 3) vs. high Foxp3 density in PD-1 positive/CD3 high density (*n* = 11). Further investigation of immune-related cells in a large number of PDAC patients adjusted to AJCC stages is needed.

To predict the prognosis of PDAC patients with R0 resection with useful markers, systemic inflammatory nutritional markers such as the NLR, PLR, LMR, CAR, and GPS were also assessed in this study. However, no good prognostic biomarkers are available. The patient number analyzed in this study may not be sufficient to detect laboratory markers associated with PDAC progression in patients with R0 resection. As the present data could be considered a very preliminary report, future studies for detection of prognostic markers using large prospective cohort studies are also recommended.

## Conclusions

PDAC patients who underwent R0 surgical resection with high TIL infiltration in the TME or PD-1+ T cells in TLAs had a significantly better prognosis than patients without these traits. Moreover, the presence of high Foxp3 infiltration in the TME was associated with poor outcomes compared to the presence of low infiltration. Overall, PDAC patients with a TME rich in TILs, PD-1+ T cells in TLAs, and low Foxp3+ T cell density may have an “immune-rich” TME, leading to a good clinical prognosis. This observation was independent of clinicopathologic parameters, suggesting a predictive role for this “immune-rich” subtype in PDAC.

## Supplementary Information

**Additional file 1.** Table S1. Base line characteristics of laboratory data and systemic inflammatory responses

**Additional file 2.** Table S2. Univariate and multivariate analysis for overall survival

**Additional file 3.** Table S3. Univariate and multivariate analysis for disease free survival

**Additional file 4.** Table S4. Association of clinicopathological characteristics with immune related cells

**Additional file 5.** Table S5. Association of laboratory parameters with immune related cells

**Additional file 6.** Table S6. Association of immune-related cells with clinical prognosis in PDAC patients with stage I/II disease

**Additional file 7.** Table S7. Association of immune-related cells with clinical prognosis in PDAC patients with stage III disease

**Additional file 8 Fig. S1.** Associations of PD-1+ cells in tertiary lymphoid aggregates with clinical prognosis

**Additional file 9 Fig. S2.** Associations of Foxp3+ cells in the PDAC TME with clinical prognosis

## Data Availability

All data generated or analyzed during this study are included in this published article.

## Abbreviations

Alb: albumin
AMY: amylase
CA19–9: carbohydrate antigen 19–9
CEA: carcinoembryonic antigen
CIs: confidence intervals
CAR: CRP/Alb ratio
CRP: C-reactive protein
CTLs: cytotoxic T lymphocytes
DCs: dendritic cells
DFS: disease-free survival
Foxp3: forkhead box P3
GPS: Glasgow prognostic score
LDH: lactate dehydrogenase
LMR: lymphocyte/monocyte ratio
MDSCs: myeloid-derived suppressor cells
NLR: neutrophil/lymphocyte ratio
OS: overall survival
PDAC: pancreatic ductal adenocarcinoma
PD-1: programmed death receptor 1
PLR: platelet/lymphocyte ratio
TAAs: tumor-associated antigens
TAMs: tumor-associated macrophages
TGF-β: tumor growth factor-β
Tregs: regulatory T cells
TILs: tumor-infiltrating lymphocytes
TLAs: tertiary lymphoid aggregates
TME: tumor microenvironment
WT1: Wilms’ tumor 1
