# Supplementary material for: Prognostic value of immune factors in the tumor microenvironment of patients with pancreatic ductal adenocarcinoma
Source: BMC Cancer. 2021 Nov 10;21:1197. doi: 10.1186/s12885-021-08911-4 (PMC8582170; doi:10.1186/s12885-021-08911-4)
Supplement: Supplementary file 1 — Additional file 1. Table S1. Base line characteristics of laboratory data and systemic inflammatory responses [file 12885_2021_8911_MOESM1_ESM.doc]

Table S1. Base line characteristics of laboratory data and systemic inflammatory responses.

|  | Median | Range |
| --- | --- | --- |
| Leucocyte (counts/µL) | 5200 | 2500-12000 |
| Lymphocyte (counts/µL) | 1400 | 500-2800 |
| Monocyte (counts/µL) | 300 | 100-900 |
| Neutrophil (counts/µL) | 3100 | 1400-9000 |
| Platelet (x10^4^ counts/µL) | 24.7 | 12.1-80.6 |
| Hemoglobin (g/dL) | 12.5 | 8.6-16.5 |
| C-reactive protein (CRP) (mg/dL) | 0.1 | 0.1-3.8 |
| Albumin (g/dL) | 3.8 | 2.0-4.9 |
| Lactate dehydrogenase (LDH) (IU/L) | 183 | 104-319 |
| Amylase (AMY) (IU/L) | 83 | 4-1556 |
| Carcinoembryonic antigen (CEA) (ng/mL) | 4.7 | 1-43.7 |
| Carbohydrate antigen 19-9 (CA19-9) (U/L) | 105 | 1-6676 |
| Neutrophil/Lymphocyte ratio (NLR) | 2.14 | 1.00-7.60 |
| Platelet/Lymphocyte ratio (PLR) | 177.7 | 61.81-424.21 |
| Lymphocyte/Monocyte ratio (LMR) | 4.75 | 0.55-11.00 |
| C-reactive protein/Albumin (CAR) | 0.03 | 0.02-1.18 |
| Glasgow prognostic score (GPS) | 0 | 0-2 |
