# Supplementary material for: Prognostic value of immune factors in the tumor microenvironment of patients with pancreatic ductal adenocarcinoma
Source: BMC Cancer. 2021 Nov 10;21:1197. doi: 10.1186/s12885-021-08911-4 (PMC8582170; doi:10.1186/s12885-021-08911-4)
Supplement: Supplementary file 2 — Additional file 2. Table S2. Univariate and multivariate analysis for overall survival [file 12885_2021_8911_MOESM2_ESM.doc]

Table S2. Univariate and multivariate analysis for overall survival.

|  | | Univariate analysis | | *p*- value | Multivariate analysis | | *p*- value |
| --- | --- | --- | --- | --- | --- | --- | --- |
|  |  | HR | 95%CI |  | HR | 95%CI |  |
| Age at surgery | per 10 years increase | 0.945 | 0.750-1.209 | 0.642 | 0.890 | 0.683-1.167 | 0.392 |
| Tumor location | Head vs. Body-to-Tail | 0.813 | 0.478-1.323 | 0.423 | 1.190 | 0.665-2.059 | 0.545 |
| Tumor differentiation | Well-to-Moderate vs. Poor | 1.833 | 0.972-3.239 | 0.047 | 0.867 | 0.436-1.632 | 0.671 |
| Tumor stage | I and II vs. III | 1.064 | 0.656-1.681 | 0.794 | 1.423 | 0.794-2.495 | 0.226 |
| Tumor size | ≥30 vs. <30 mm | 1.252 | 0.778-2.084 | 0.368 | 1.129 | 0.663-1.967 | 0.660 |
| Lymphocyte counts | per 100 increase | 1.004 | 0.958-1.050 | 0.880 | 0.988 | 0.930-1.045 | 0.673 |
| Neutrophil/Lymphocyte ratio (NLR) | per 1 increase | 0.936 | 0.784-1.100 | 0.441 | 1.135 | 0.902-1.407 | 0.260 |
| Glasgow prognostic score (GPS) | per 1 increase | 1.342 | 0.884-1.946 | 0.142 | 0.897 | 0.528-1.475 | 0.677 |
| CD3+ T cell density | High vs. Low | 0.124 | 0.070-0.215 | <0.001 | - | | |
| CD4+ T cell density | High vs. Low | 0.167 | 0.092-0.290 | <0.001 | - | | |
| CD8+ T cell density | High vs. Low | 0.279 | 0.173-0.445 | <0.001 | - | | |
| PD-1+ T cell positivity | Positive vs. Negative | 0.338 | 0.198-0.557 | <0.001 | - | | |
| Foxp3+ T cell density | High vs. Low | 2.107 | 1.322-3.346 | 0.002 | - | | |
| Local expression pattern | CD3=Low | 1.000 | reference |  | 1.000 | reference |  |
|  | CD3=High, PD-1=Negative | 0.187 | 0.101-0.337 | <0.001 | 0.136 | 0.064-0.278 | <0.001 |
|  | CD3=High, PD-1=Positive, Foxp3=High | 0.182 | 0.060-0.443 | <0.001 | 0.139 | 0.043-0.371 | <0.001 |
|  | CD3=High, PD-1=Positive, Foxp3=Low | 0.054 | 0.023-0.117 | <0.001 | 0.035 | 0.013-0085 | <0.001 |

HR: hazard ratio, CI: confidence interval
