# Supplementary material for: Prognostic value of immune factors in the tumor microenvironment of patients with pancreatic ductal adenocarcinoma
Source: BMC Cancer. 2021 Nov 10;21:1197. doi: 10.1186/s12885-021-08911-4 (PMC8582170; doi:10.1186/s12885-021-08911-4)
Supplement: Supplementary file 3 — Additional file 3. Table S3. Univariate and multivariate analysis for disease free survival [file 12885_2021_8911_MOESM3_ESM.doc]

Table S3. Univariate and multivariate analysis for disease free survival.

|  | | Univariate analysis | | *p*- value | Multivariate analysis | | *p*- value |
| --- | --- | --- | --- | --- | --- | --- | --- |
|  |  | HR | 95%CI |  | HR | 95%CI |  |
| Age at surgery | per 10 years increase | 0.986 | 0.789-1.251 | 0.905 | 0.918 | 0.715-1.193 | 0.514 |
| Tumor location | Head vs. Body-to-Tail | 1.006 | 0.598-1.625 | 0.981 | 1.544 | 0.872-2.664 | 0.125 |
| Tumor differentiation | Well-to-Moderate vs. Poor | 2.006 | 1.062-3.549 | 0.023 | 1.010 | 0.501-1.940 | 0.976 |
| Tumor stage | I and II vs. III | 0.851 | 0.525-1.341 | 0.497 | 0.757 | 0.431-1.296 | 0.321 |
| Tumor size | <30 mm vs. ≥30 mm | 1.323 | 0.826-2.191 | 0.259 | 1.091 | 0.648-1.887 | 0.748 |
| Lymphocyte counts | per 100 increase | 1.007 | 0.960-1.055 | 0.773 | 1.024 | 0.963-1.087 | 0.440 |
| Neutrophil/Lymphocyte ratio (NLR) | per 1 increase | 0.972 | 0.819-1.136 | 0.732 | 1.198 | 0.955-1.483 | 0.106 |
| Glasgow prognostic score (GPS) | per 1 increase | 1.211 | 0.787-1.783 | 0.356 | 1.042 | 0.588-1.796 | 0.886 |
| CD3+ T cell density | High vs. Low | 0.204 | 0.118-0.345 | <0.001 | - | | |
| CD4+ T cell density | High vs. Low | 0.249 | 0.145-0.418 | <0.001 | - | | |
| CD8+ T cell density | High vs. Low | 0.331 | 0.205-0.526 | <0.001 | - | | |
| PD-1+ T cell positivity | Positive vs. Negative | 0.376 | 0.221-0.620 | <0.001 | - | | |
| Foxp3+ T cell density | High vs. Low | 2.270 | 1.426-3.606 | <0.001 | - | | |
| Local expression pattern | CD3=Low | 1.000 | reference |  | 1.000 | reference |  |
|  | CD3=High, PD-1=Negative | 0.293 | 0.160-0.518 | <0.001 | 0.245 | 0.120-0.479 | <0.001 |
|  | CD3=High, PD-1=Positive, Foxp3=High | 0.336 | 0.114-0.797 | 0.025 | 0.326 | 0.106-0.815 | 0.028 |
|  | CD3=High, PD-1=Positive, Foxp3=Low | 0.089 | 0.038-0.191 | <0.001 | 0.065 | 0.024-0.155 | <0.001 |

HR: hazard ratio, CI: confidence interval
