# Supplementary material for: Prognostic value of immune factors in the tumor microenvironment of patients with pancreatic ductal adenocarcinoma
Source: BMC Cancer. 2021 Nov 10;21:1197. doi: 10.1186/s12885-021-08911-4 (PMC8582170; doi:10.1186/s12885-021-08911-4)
Supplement: Supplementary file 4 — Additional file 4. Table S4. Association of clinicopathological characteristics with immune related cells [file 12885_2021_8911_MOESM4_ESM.doc]

Table S4. Association of clinicopathological characteristics with immune related cells.

| Clinicopathological characteristics | | CD3+ T cell density | | *P*-value | CD4+ T cell density | | *P*-value | CD8+ T cell density | | *P*-value | PD-1+ T cell positivity | | *P*-value | Foxp3+ T cell density | | *P*-value |
| --- | --- | --- | --- | --- | --- | --- | --- | --- | --- | --- | --- | --- | --- | --- | --- | --- |
|  |  | Low | High |  | Low | High |  | Low | High |  | Negative | Positive |  | Low | High |  |
| Age at surgery | ≥median (67 years) | 22(50.0%) | 26(63.4%) | 0.213 | 26(54.2%) | 22(59.5%) | 0.626 | 27(55.1%) | 21(58.3%) | 0.767 | 35(61.4%) | 13(46.4%) | 0.191 | 26(53.1%) | 22(61.1%) | 0.460 |
|  | <median | 22(50.0%) | 15(36.6%) |  | 22(45.8%) | 15(40.5%) |  | 22(44.9%) | 15(41.7%) |  | 22(38.6%) | 15(53.6%) |  | 23(46.9%) | 14(38.9%) |  |
| Sex | Male | 25(56.8%) | 22(53.7%) | 0.770 | 26(54.2%) | 21(56.8%) | 0.812 | 26(53.1%) | 21(58.3%) | 0.629 | 35(61.4%) | 12(42.9%) | 0.106 | 31(63.3%) | 16(44.4%) | 0.085 |
|  | Female | 19(43.2%) | 19(46.3%) |  | 22(45.8%) | 16(43.2%) |  | 23(46.9%) | 15(41.7%) |  | 22(38.6%) | 16(57.1%) |  | 18(36.7%) | 20(55.6%) |  |
| Tumor location | Head | 36(81.8%) | 28(68.3%) | 0.149 | 37(77.1%) | 27(73.0%) | 0.663 | 38(77.6%) | 26(72.2%) | 0.574 | 43(75.4%) | 21(75.0%) | 0.965 | 35(61.4%) | 29(80.6%) | 0.335 |
|  | Body-to-Tail | 8(18.2%) | 13(31.7%) |  | 11(28.9%) | 10(27.0%) |  | 11(22.4%) | 10(27.8%) |  | 14(24.6%) | 7(25.0%) |  | 14(28.6%) | 7(19.4%) |  |
| Tumor differentiation | Well-to-Moderate | 34(77.3%) | 37(90.0%) | 0.107 | 37(77.1%) | 34(91.9%) | 0.068 | 38(77.6%) | 33(71.7%) | 0．083 | 45(78.9%) | 26(92.9%) | 0.104 | 42(85.7%) | 29(80.6%) | 0.526 |
|  | Poor | 10(22.7%) | 4(10.0%) |  | 11(28.9%) | 3(8.1%) |  | 11(22.4%) | 3(8.3%) |  | 12(21.1%) | 2(7.1%) |  | 7(14.3%) | 7(19.4%) |  |
| Tumor stage | I and II | 31(70.5%) | 28(68.3%) | 0.829 | 30(62.5%) | 29(78.4%) | 0.115 | 31(63.2%) | 28(77.8%) | 0.151 | 40(70.2%) | 19(67.9%) | 0.827 | 31(63.3%) | 28(77.8%) | 0.151 |
|  | III | 13(29.5%) | 13(31.7%) |  | 18(37.5%) | 8(21.6%) |  | 18(36.8%) | 8(22.2%) |  | 17(29.8%) | 9(32.1%) |  | 18(36.7%) | 8(22.2%) |  |
| Tumor size | ≥median (35 mm) | 22(50.0%) | 23(56.1%) | 0.574 | 28(58.3%) | 17(45.9%) | 0.257 | 28(57.1%) | 17(47.2%) | 0.365 | 31(54.4%) | 14(50.0%) | 0.703 | 28(57.1%) | 17(47.2%) | 0.365 |
|  | <median | 22(50.0%) | 18(43.9%) |  | 20(41.7%) | 20(54.1%) |  | 21(42.9%) | 19(52.8%) |  | 26(45.6%) | 14(50.0%) |  | 21(42.9%) | 19(52.8%) |  |

All data shows n (%).
