# Supplementary material for: Prognostic value of immune factors in the tumor microenvironment of patients with pancreatic ductal adenocarcinoma
Source: BMC Cancer. 2021 Nov 10;21:1197. doi: 10.1186/s12885-021-08911-4 (PMC8582170; doi:10.1186/s12885-021-08911-4)
Supplement: Supplementary file 5 — Additional file 5. Table S5. Association of laboratory parameters with immune related cells [file 12885_2021_8911_MOESM5_ESM.doc]

Table S5. Association of laboratory parameters with immune related cells.

|  | CD3+ T cell density | | *P*-value | CD4+ T cell density | | *P*-value | CD8+ T cell density | | *P*-value | PD-1+ T cell positivity | | *P*-value | Foxp3+ T cell density | | *P*-value |
| --- | --- | --- | --- | --- | --- | --- | --- | --- | --- | --- | --- | --- | --- | --- | --- |
|  | Low | High |  | Low | High |  | Low | High |  | Negative | Positive |  | Low | High |  |
| Leucocyte (counts/μL) | 5050(3975-6025) | 5300(4600-6400) | 0.289 | 5050(4175-5825) | 5400(4500-6500) | 0.378 | 5000(4100-5800) | 5500(4675-6425) | 0.166 | 5200(4000-6100) | 5200(4956-6001) | 0.513 | 5100(4300-6400) | 5300(4350-6025) | 0.735 |
| Lymphocyte (counts/μL) | 1499(1100-1750) | 1400(1200-1700) | 0.958 | 1350(1100-1700) | 1400(1100-1700) | 0.505 | 1400(1200-1700) | 1400(1100-1700) | 0.740 | 1400(1100-1700) | 1450(1313-1644.1) | 0.789 | 1300(1100-1700) | 1500(1300-1750) | 0.086 |
| Monocyte (counts/μL) | 300(200-400) | 300(200-400) | 0.938 | 300(200-400) | 300(200-400) | 0.890 | 300(200-400) | 300(200-400) | 0.083 | 300(200-400) | 300(273-344) | 0.873 | 300(200-300) | 300(300-400) | 0.121 |
| Neutrophil (counts/μL) | 3000(2300-4050) | 3300(2600-4400) | 0.218 | 3050(2500-3850) | 3360(2400-4500) | 0.432 | 3000(2400-3800) | 3500(2650-4500) | 0.280 | 3100(2400-4200) | 3250(3031.1-3587.7) | 0.443 | 3200(2300-4400) | 3100(2500-3850) | 0.873 |
| Platelet (×10^4^ counts/μL) | 24.20(19.18-28.65) | 21.7(16.40-29.00) | 0.344 | 25.15(18.15-28.88) | 21.20(16.20-28.50) | 0.425 | 24.60(18.20-28.50) | 21.45(16.18-29.05) | 0.893 | 22.30(17.60-27.10) | 26.90(17.15-29.18) | 0.246 | 22.30(17.10-28.50) | 22.75(17.98-29.28) | 0.491 |
| Hemoglobin (g/dL) | 12.5(11.7-13.9) | 12.6(12.0-13.9) | 0.654 | 12.5(11.9-13.6) | 12.6(11.3-14.0) | 0.989 | 12.6(12.0-13.9) | 12.3(11.3-13.9) | 0.100 | 12.5(11.8-13.9) | 12.6(11.8-13.9) | 0.841 | 13.0(12.0-14.0) | 12.2(11.7-13.2) | 0.118 |
| C-reactive protein (CRP) (mg/dL) | 0.17(0.1-0.33) | 0.10(0.10-0.25) | 0.187 | 0.13(0.10-0.30) | 0.10(0.10-0.30) | 0.468 | 0.1(0.1-0.3) | 0.1(0.1-0.3) | 0.996 | 0.10(0.10-0.30) | 0.10(0.10-0.26) | 0.329 | 0.10(0.10-0.60) | 0.10(0.10-0.26) | 0.696 |
| Albumin (g/dL) | 3.8(3.5-4.1) | 3.9(3.6-4.2) | 0.505 | 3.8(3.4-4.1) | 3.9(3.8-4.2) | 0.230 | 3.8(3.5-4.2) | 3.9(3.6-4.1) | 0.471 | 3.9(3.6-4.2) | 3.8(3.6-4.1) | 0.59 | 3.9(3.6-4.1) | 3.8(3.6-4.2) | 0.803 |
| Lactate dehydrogenase (LDH) (IU/L) | 183.5(166.3-204.5) | 180.0(155.0-204.0) | 0.553 | 185.0(161.2-212.0) | 175.0(157.0-191.0) | 0.412 | 183.0(159.0-206.0) | 179.5(157.8-197.8) | 0.879 | 185.0(162.0-210.0) | 176.5(155.0-195.8) | 0.35 | 182.0(155.0-210.0) | 185.0(166.3-198.8) | 0.516 |
| Amylase (AMY) (IU/L) | 86.5(44.3-152.8) | 83.0(56.0-128.0) | 0.866 | 72.0(33.0-115.0) | 108(61-154) | 0.069 | 78.0(33.0-115.0) | 115.0(59.0-186.5) | 0.172 | 80.0(54.0-147.0) | 87.0(58.3-141.3) | 0.9 | 81.5(51.8-156.5) | 91.0(56.0-124.5) | 0.843 |
| Carcinoembryonic antigen (CEA) (ng/mL) | 5.15(3.10-8.65) | 4.50(2.00-6.70) | 0.508 | 5.45(3.13-8.35) | 4.5(2.80-6.25) | 0.260 | 4.6(3.0-8.8) | 2.7(3.3-6.4) | 0.695 | 4.50(3.03-6.53) | 5.60(3.15-9.00) | 0.538 | 5.25(3.48-8.23) | 4.10(2.50-6.80) | 0.19 |
| Carbohydrate antigen 19- 9 (CA19-9) (U/mL) | 153(47-348) | 69(29-202) | 0.192 | 125.0(53.0-351.0) | 81.0(28.75-211.5) | 0.194 | 125(49-345) | 77.5(32.0-314.3) | 0.375 | 113(49-347) | 64(24-212) | 0.516 | 93(31-278) | 117(52-351) | 0.284 |
| Neutrophil/Lymphocyte ratio (NLR) | 2.143(1.778-2.475) | 2.168(1.636-3.385) | 0.527 | 2.160(1.746-2.746) | 2.091(1.643-3.296) | 0.947 | 2.143(1.769-2.500) | 2.307(1.627-3.404) | 0.758 | 2.143(1.750-3.143) | 2.200(2.103-3.008) | 0.881 | 2.167(1.778-3.385) | 2.077(1.622-2.597) | 0.35 |
| Platelets/Lymphocyte ratio (PLR) | 174.08(133.74-210.52) | 167.65(127.50-214.12) | 0.574 | 176.20(135.17-217.45) | 167.27(127.50-196.0) | 0.240 | 173.16(132.94-212.50) | 167.91(130.0-214.70) | 0.918 | 168.18(127.50-212.50) | 173.25(158.46-197.64) | 0.472 | 172.86(135.56-212.50) | 167.79(125.63-214.68) | 0.581 |
| Lymphocyte/Monocyte ratio (LMR) | 4.833(3.625-6.542) | 4.750(4.000-6.333) | 0.933 | 4.33(3.50-6.27) | 5.00(4.00-6.67) | 0.579 | 5.0(4.0-6.33) | 4.708(3.375-6.375) | 0.323 | 5.000(3.500-6.3330) | 4.583(4.362-5.995) | 0.797 | 5.000(3.400-6.500) | 4.400(4.000-6.083) | 0.862 |
| C-reactive protein/Albumin (CAR) | 0.0393(0.0261-0.0994) | 0.0278(0.0244-0.0758) | 0.133 | 0.0337(0.263-0.0835) | 0.0278(0.0238-0.0789) | 0.139 | 0.0333(0.0263-0.0810) | 0.0282(0.0249-0.0789) | 0.929 | 0.0333(0.0250-0.0811) | 0.0278(0.0248-0.0766) | 0.546 | 0.0294(0.0250-0.1290) | 0.0313(0.0255-0.7660) | 0.732 |
| Glasgow prognostic score (GPS) | 0(0-1) | 0(0-0) | 0.078 | 0(0-1) | 0(0-0) | 0.026 | 0(0-1) | 0(0-0) | 0.308 | 0(0-1) | 0(0-0) | 0.259 | 0(0-0) | 0(0-1) | 0.521 |

All data show median (Interquartile range min-max).
