# Supplementary material for: Prognostic value of immune factors in the tumor microenvironment of patients with pancreatic ductal adenocarcinoma
Source: BMC Cancer. 2021 Nov 10;21:1197. doi: 10.1186/s12885-021-08911-4 (PMC8582170; doi:10.1186/s12885-021-08911-4)
Supplement: Supplementary file 6 — Additional file 6. Table S6. Association of immune-related cells with clinical prognosis in PDAC patients with stage I/II disease [file 12885_2021_8911_MOESM6_ESM.doc]

Table S6. Association of immune-related cells with clinical prognosis in PDAC patients with stage I/II disease.

|  | | Patient numbers | median OS (days) | *p*-value | median DFS (days) | *p*-value |
| --- | --- | --- | --- | --- | --- | --- |
| CD3+ T cell density | High vs. Low | 28 vs. 31 | 774 vs. 400 | <0.0001 | 394.5 vs. 174 | <0.0001 |
| CD4+ T cell density | High vs. Low | 30 vs. 29 | 773.5 vs. 424 | <0.0001 | 380.5 vs. 184 | <0.0001 |
| CD8+ T cell density | High vs. Low | 26 vs. 33 | 773.5 vs. 424 | <0.0001 | 380.5 vs. 184 | 0.0006 |
| PD-1+ T cell positivity | Positive vs. Negative | 19 vs. 40 | 792 vs. 471 | 0.0003 | 391 vs. 222 | 0.0039 |
| Foxp3+ T cell density | High vs. Low | 28 vs. 31 | 428.5 vs. 615 | 0.0015 | 174.5 vs. 333 | 0.0009 |
| PD-1 expression in CD3+ T cell high density | Positive vs. Negative | 14 vs. 14 | 1223 vs. 640.5 | 0.0040 | 533.5 vs. 351 | 0.0323 |
| Foxp3 density in CD3+ T cell high density | High vs. Low | 8 vs. 20 | 563 vs. 1009.5 | 0.0001 | 237.5 vs. 510 | 0.0016 |
| Foxp3 density in PD-1 positive/CD3+ T cell high density | High vs. Low | 3 vs. 11 | 572 vs. 1360 | 0.1373 | 284 vs. 760 | 0.0586 |

PDAC, pancreatic ductal adenocarcinoma
