# Supplementary material for: Prognostic value of immune factors in the tumor microenvironment of patients with pancreatic ductal adenocarcinoma
Source: BMC Cancer. 2021 Nov 10;21:1197. doi: 10.1186/s12885-021-08911-4 (PMC8582170; doi:10.1186/s12885-021-08911-4)
Supplement: Supplementary file 7 — Additional file 7. Table S7. Association of immune-related cells with clinical prognosis in PDAC patients with stage III disease [file 12885_2021_8911_MOESM7_ESM.doc]

Table S7. Association of immune-related cells with clinical prognosis in PDAC patients with stage III disease.

|  | | Patient numbers | median OS (days) | *p*-value | median DFS (days) | *p*-value |
| --- | --- | --- | --- | --- | --- | --- |
| CD3+ T cell density | High vs. Low | 13 vs. 13 | 759 vs. 320 | <0.0001 | 587 vs. 196 | 0.0001 |
| CD4+ T cell density | High vs. Low | 8 vs. 18 | 1380.5 vs. 396 | 0.0002 | 723.5 vs. 207 | 0.0010 |
| CD8+ T cell density | High vs. Low | 10 vs. 16 | 921 vs. 396 | 0.0007 | 643 vs. 207 | 0.0006 |
| PD-1+ T cell positivity | Positive vs. Negative | 9 vs. 17 | 729 vs. 431 | 0.0561 | 484 vs. 196 | 0.0097 |
| Foxp3+ T cell density | High vs. Low | 16 vs. 10 | 450 vs. 531 | 0.7421 | 208.5 vs. 309.5 | 0.4496 |
| PD-1 expression in CD3+ T cell high density | Positive vs. Negative | 7 vs. 6 | 963 vs. 749 | 0.4871 | 660 vs. 446 | 0.1570 |
| Foxp3 density in CD3+ T cell high density | High vs. Low | 5 vs. 8 | 692 vs. 1320.5 | 0.0228 | 324 vs. 704 | 0.0726 |
| Foxp3 density in PD-1 positive/CD3+ T cell high density | High vs. Low | 2 vs. 5 | 710.5 vs. 1678 | 0.1230 | 492 vs. 821 | 0.1865 |

PDAC, pancreatic ductal adenocarcinoma
