# Supplementary material for: Prognostic value of immune factors in the tumor microenvironment of patients with pancreatic ductal adenocarcinoma
Source: BMC Cancer. 2021 Nov 10;21:1197. doi: 10.1186/s12885-021-08911-4 (PMC8582170; doi:10.1186/s12885-021-08911-4)
Supplement: Supplementary file 8 — Additional file 8 Fig. S1. Associations of PD-1+ cells in tertiary lymphoid aggregates with clinical prognosis [file 12885_2021_8911_MOESM8_ESM.doc]

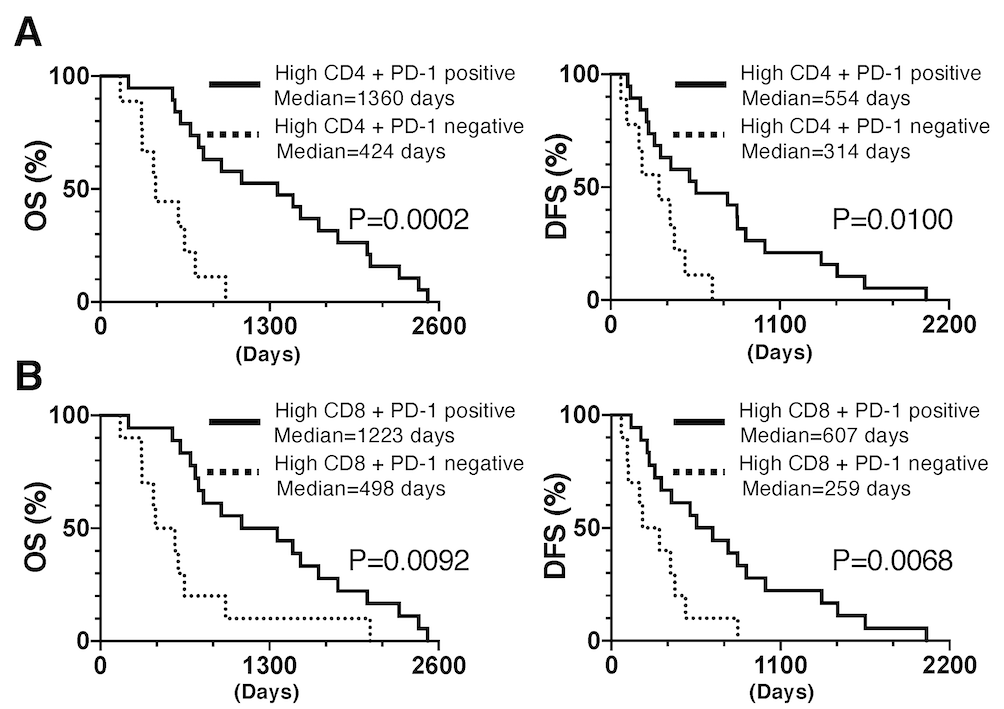


**Figure S1.** Associations of PD-1+ cells in tertiary lymphoid aggregates with clinical prognosis.

The Kaplan-Meier estimates of the OS (left panel) and DFS (right panel) times of the 2 subgroups are shown. (A) PDAC patients with high CD4+ T cell density were classified into 2 subgroups: high CD4+ T cell density/PD-1-positive T cells and high CD4+ T cell density/PD-1-negative T cells. (B) PDAC patients with high CD8+ T cell density were classified into 2 subgroups: high CD8+ T cell density/PD-1-positive T cells and high CD8+ T cell density/PD-1-negative T cells. PD-1: programmed death 1; PDAC: pancreatic ductal adenocarcinoma; OS: overall survival; DFS: disease-free survival
